# Single-cell transcriptional landscape of long non-coding RNAs orchestrating mouse heart development

**DOI:** 10.1038/s41419-023-06296-9

**Published:** 2023-12-18

**Authors:** Thaís A. R. Ramos, Sebastián Urquiza-Zurich, Soo Young Kim, Thomas G. Gillette, Joseph A. Hill, Sergio Lavandero, Thaís G. do Rêgo, Vinicius Maracaja-Coutinho

**Affiliations:** 1 Advanced Center for Chronic Diseases (ACCDiS), Faculty of Chemical & Pharmaceutical Sciences & Faculty of Medicine, Universidad de Chile, Santiago, Chile; 2 Programa de Pós-Graduação em Bioinformática, Bioinformatics Multidisciplinary Environment (BioME), Instituto Metrópole Digital, Universidade Federal do Rio Grande do Norte, João Pessoa, Brazil; 3 Departamento de Informática, Centro de Informática, Universidade Federal da Paraíba, João Pessoa, Brazil; 4 Division of Cardiology, Department of Internal Medicine, University of Texas Southwestern Medical Center Dallas, Dallas, TX USA; 5 Department of Molecular Biology, University of Texas Southwestern Medical Center, Dallas, TX USA; 6 Corporación Centro de Estudios Científicos de las Enfermedades Crónicas (CECEC), Santiago, Chile

**Keywords:** Genetics research, Differentiation, Heart failure

## Abstract

Long non-coding RNAs (lncRNAs) comprise the most representative transcriptional units of the mammalian genome. They are associated with organ development linked with the emergence of cardiovascular diseases. We used bioinformatic approaches, machine learning algorithms, systems biology analyses, and statistical techniques to define co-expression modules linked to heart development and cardiovascular diseases. We also uncovered differentially expressed transcripts in subpopulations of cardiomyocytes. Finally, from this work, we were able to identify eight cardiac cell-types; several new coding, lncRNA, and pcRNA markers; two cardiomyocyte subpopulations at four different time points (ventricle E9.5, left ventricle E11.5, right ventricle E14.5 and left atrium P0) that harbored co-expressed gene modules enriched in mitochondrial, heart development and cardiovascular diseases. Our results evidence the role of particular lncRNAs in heart development and highlight the usage of co-expression modular approaches in the cell-type functional definition.

## Introduction

Approximately 90% of the mammalian genome is transcribed in a cell-specific manner into RNA [1], but only 2–3% is transcribed to protein-coding messenger RNA (mRNA) [2, 3]. Most RNA transcripts are classified as non-coding RNAs (ncRNAs) and can be divided into small (sncRNAs) and long ncRNAs (lncRNAs) [2–5]. These ncRNAs play a crucial role in controlling the flow of genetic information through various gene regulation mechanisms [5, 6], LncRNAs exhibit cell type- and tissue-specific expression and have the ability to influence lineage fates and cell subtypes [7, 8]. Although the involvement of ncRNAs in cardiovascular diseases has been extensively studied [3, 6, 9, 10], our understanding of the expression and function of lncRNAs in heart development remains limited [11]. Investigating the single-cell expression patterns of lncRNAs could unveil novel biological functions associated with different cell populations. Therefore, accurately measuring the abundances of lncRNAs in individual cells is critical for identifying cell-type specificities and determining single-cell functions [12].

A new type of lncRNA, known as positionally conserved lncRNAs (pcRNAs), has been identified based on the evolutionary conservation of their promoters and their syntenic positions between humans and mice [6]. These pcRNAs are apparently involved in developing cardiovascular tissue, arterial morphogenesis, and smooth muscle tissue cell differentiation. A subset of them manifested potential importance in chromatin organization, as evidenced by their localization overlapping with the binding sites for the CTCF chromatin organizer, the anchor points of chromatin loops, and the borders of topologically associating domains [6].

The failing adult heart resembles the fetal heart, through the ‘re-expression of the fetal gene expression program’ [13, 14]. Our study aims to dissect patterns and cell-specific distributions of coding and non-coding RNAs during development using single-cell data. This information will enable us to unveil novel regulatory processes occurring during cardiac development and potentially lead to improved therapeutic targeting [2–4].

## Results

### Expression quantification and cell-type classification

**Fig. 1:**
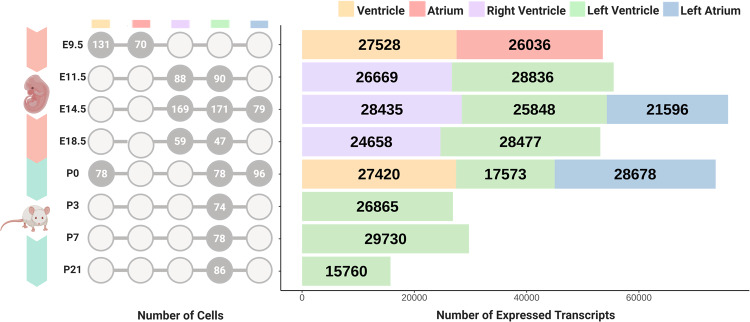
Single-cell transcriptomics dataset based on the number of cells and transcripts in each myocardial compartment across developmental stages. Circular diagram reports the number of single cells for each data point. The bar graph shows the number of expressed transcripts color-coded by anatomical area (yellow: ventricle, red: atrium, purple: right ventricle, green: left ventricle, blue: left atrium). The left ventricle was the only compartment with data collected across all time points.

We investigated the gene expression patterns during cardiac development using single-cell RNA sequencing (scRNA-seq) data. The dataset encompassed four embryonic and four postnatal stages, covering five distinct myocardial anatomical areas (Fig. 1). Employing a bioinformatic pipeline (See Supplementary Methods), we retrieved the expression levels of all transcripts normalized using FPKM values, requiring that they must be expressed in at least 5% of cells to be considered in subsequent analyses. Table 1 shows the expression profiles of coding and long non-coding transcripts identified in specific compartments and time-points. The number of lncRNAs ranged from 738 (left ventricle P21) to 2,636 (left ventricle P7) transcripts, with P21 exhibiting fewer than 1000. These findings aligned with previously reported studies related to numbers of lncRNAs in breast cancer, cardiac fibrosis, and heart development [15–18].

**Table 1:** Number of transcripts expressed in Fig. 1, marker transcripts (coding genes, lncRNAs and pcRNAs) and distribution in each compartment according to development stage.

Stage	Compartment	Coding genes		lncRNAs		pcRNAs	
		Total	Markers	Total	Markers	Total	Markers
E9.5	Ventricle	25217	1770	2190	186	121	13
E9.5	Atrium	24181	453	1751	27	104	4
E11.5	Right ventricle	25056	1432	1514	96	99	11
E11.5	Left ventricle	26468	1767	2246	241	122	11
E14.5	Right ventricle	25826	2939	2467	448	142	24
E14.5	Left ventricle	23929	1446	1814	154	105	14
E14.5	Left atrium	20077	329	1438	17	81	3
E18.5	Right ventricle	22771	221	1786	24	101	0
E18.5	Left ventricle	26150	387	2194	38	133	5
P0	Ventricle	25428	673	1880	70	112	6
P0	Left ventricle	16453	63	1064	7	56	2
P0	Left atrium	26350	757	2194	122	134	10
P3	Left ventricle	24582	632	2174	68	109	2
P7	Left ventricle	26957	574	2636	60	137	2
P21	Left ventricle	14984	67	738	6	38	0

Expressed transcripts and marker transcripts are reported according to each of the three classes (coding, lncRNAs, and pcRNAs) in each compartment across developmental stages. The first column is the development stage; the second column represents the compartment; 3rd and 4th columns are related to the number of expressed coding genes and markers coding genes, respectively; 5th and 6th columns report expressed lncRNAs and markers lncRNAs, respectively; finally, 7th and 8th columns reported expressed pcRNAs and markers pcRNAs, respectively.

**Fig. 2:**
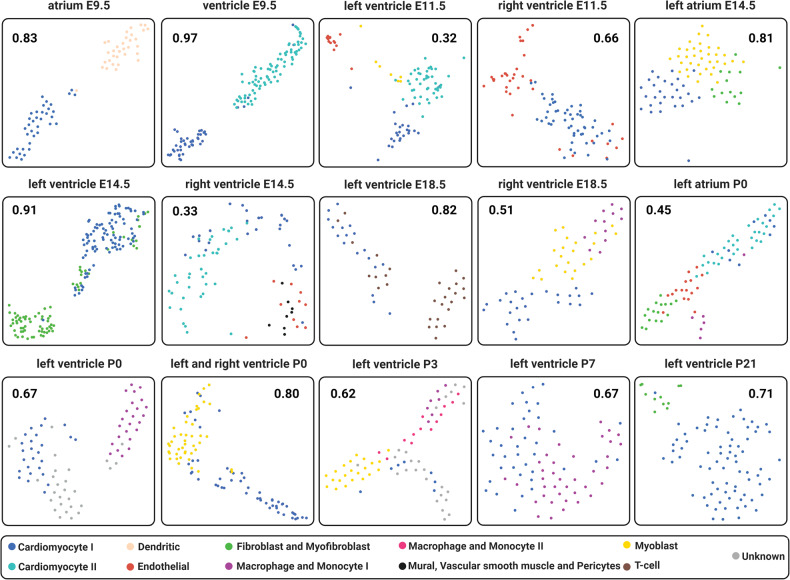
Representative cell-type assignment in each anatomical area per time point. t-SNE plots in which each square represents a compartment at a specific development stage. Each dot represents a cell, and each color describes a particular cell-type: Blue - Cardiomyocyte I; Light blue - Cardiomyocyte II; Salmon - Dendritic; Red - Endothelial; Green - Fibroblast and Myofibroblast; Purple - Macrophage and Monocyte II; Black - Mural, Vascular smooth muscle and Pericytes; Myoblast - Yellow; Brown - T-cell; and Gray - Unknown cell-types (i.e. can be a different cell-type in which we don’t have markers). In addition, each number inside the squares is related to the silhouette coefficient score for each compartment and time point.

To characterize the cell types and assign them based on expression values, we performed cluster analysis. The Silhouette method [19] combined with hierarchical clustering determined the optimal number of clusters for each sample. Chi-squared and adherence tests assessed cluster significance and assigned cell types. Cluster visualization was achieved through t-SNE plots for identification of different cell populations, illustrating the spatial distribution of cell types based on transcript expression patterns (Fig. 2). t-SNE uses a Gaussian probability function to calculate how likely a cell will pick another cell as its neighbor and repeats this step for all cells. Heatmaps (Supplementary Fig. 1) visually represented expression patterns by cell type, confirming t-SNE cell assignments (Fig. 2). Postnatal stages revealed a maximum of five potential cell clusters, while embryonic ventricle stages showed four clusters. Supplementary File 1 contains a list of potential gene markers identified based on expression patterns.

Then, we classified clusters into specific cell types based on the expression patterns of known marker transcripts, integrating information from three [20–22] and the PanglaoDB database [23], creating a comprehensive heart cell marker database. M3Drop [24], fitting a Michaelis-Menten model, identified significantly variable genes that were expressed among the cell clusters, which were then cross-referenced with our heart cell markers database. Chi-squared and adherence tests determined cluster assignments significance. Eight distinct cell-types were identified in all compartments and time-points: cardiomyocytes, myoblasts, endothelial cells, vascular smooth muscle cells and pericytes, fibroblasts and myofibroblasts, T-cells, macrophages, monocytes and dendritic cells (Fig. 2). Four compartments and time-points exhibited two subpopulations of cardiomyocytes, and left ventricle P3 showed two macrophages and monocyte subpopulations. Supplementary File 1 provides markers identified for each cell-type at each stage. Supplementary Fig. 1 presents heatmaps and hierarchical clusters of the expression patterns for each heart chamber and time-point, corroborating each subtype assignment.

### scRNA-seq reveals lncRNAs as potential cell markers for eight cell-types during heart development

**Fig. 3:**
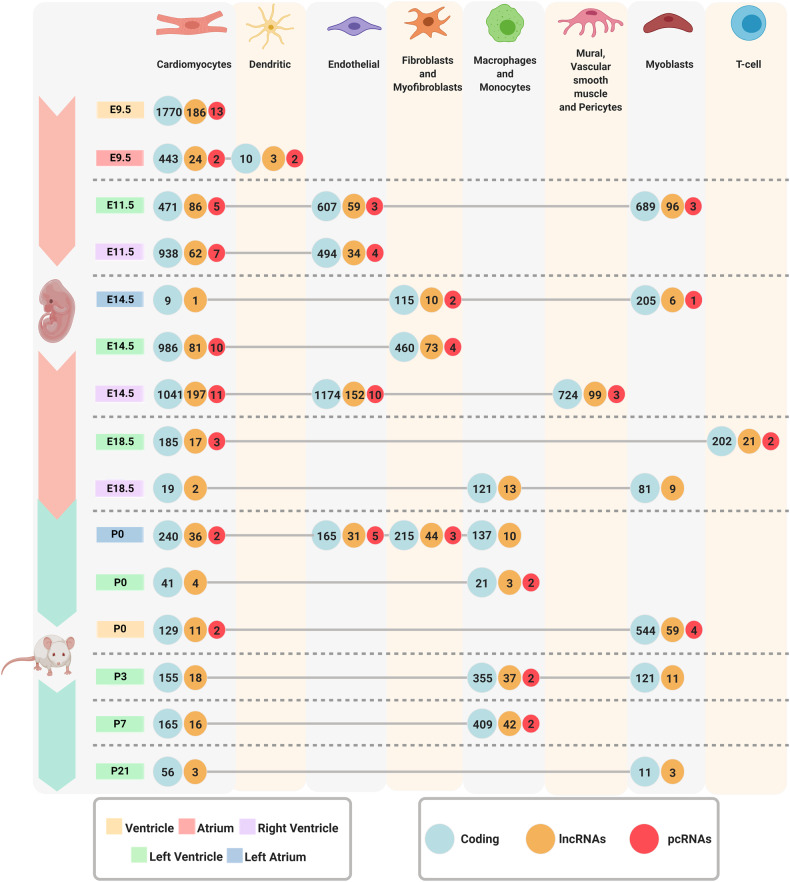
Cell marker transcripts by cell-type and chamber during heart development. The numbers inside each color-coded circle (green: coding genes, orange: lncRNAs, red: pcRNAs) represent the number of marker’s transcripts. Each compartment is represented by a color: Yellow - Ventricle; Red - Atrium; Purple - Right Ventricle; Green - Left Ventricle; and Blue - Left Atrium. In addition, we found marker’s through 8 different cell-types represented by each column.

The transcript cell markers determined above were divided into three classes, including coding mRNAs, lncRNAs, and pcRNAs (Fig. 3, Supplementary File 1), which were further explored to analyze their potential involvement in the heterogeneity of cell populations across various time points and chambers during heart development. PcRNAs served as markers for multiple cell-types. We found at least one lncRNA (cardiomyocytes, left atrium E14.5) as a cell marker in all time-points and chambers, reaching up to 197 in cardiomyocytes of the right ventricle E14.5. Our data confirmed the presence of cardiomyocytes in all compartments at all development stages [20–23, 25, 26], while also revealing region- and developmental stage-specific cell populations. For instance, a cell population with a dendritic cell-like transcriptional signature was observed exclusively in E9.5 atrium; meanwhile, we identified a vascular smooth muscle-like population in E14.5 right ventricle and a T cell-like population in E18.5 left ventricle. Interestingly, fibroblasts and myofibroblast transcriptional signatures were detected in E14.5, a stage at which fibroblasts from the epicardium are known to undergo epithelial-to-mesenchymal transition (EMT) [26]. Cell populations manifesting monocyte and macrophage profiles appeared only at late embryonic and postnatal stages, matching previously reported evidence [27]. Here, findings highlight the relevance of different lncRNAs and pcRNAs signatures in region- and stage-specific cellular populations during heart development.

We manually explored the cell types that can be identified in the same anatomical region (e.g. fibroblasts and myofibroblasts in the left atrium) that were previously reported [28]. At the E9.5, non-fibroblast-enriched cells were identified in atrium or ventricle compartments, consistent with a prior report [20]. We argue that our cell-type analysis based on coding and lncRNA expression patterns may reflect unique aspects of myocardial developmental events. In addition, it not only matches the analysis based on the coding transcriptome alone [20] but also reveals new cell populations not uncovered in that analysis (Fig. 3). These results were consistent with the previous report but also provided a novel insight by uncovering additional cell types that were not previously reported, offering a comprehensive understanding of the cellular composition under heart development.

### scRNA-seq identifies specific signatures for coding genes and lncRNAs for the same cell-type at different heart compartments

**Fig. 4:**
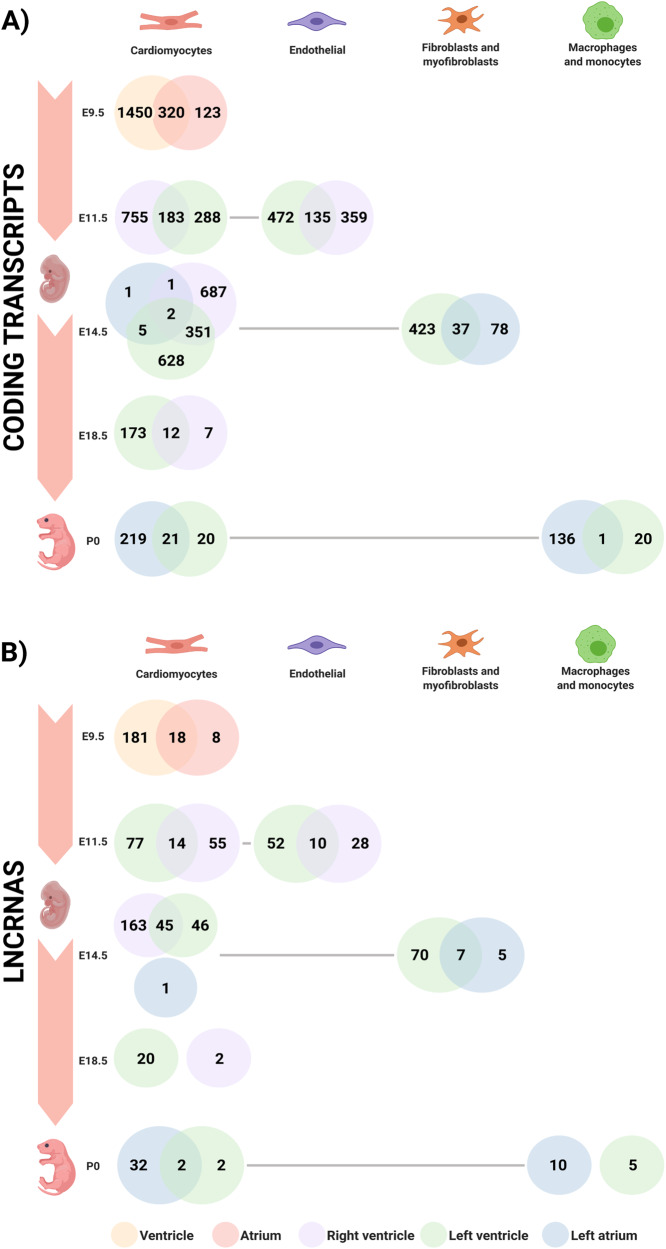
Venn diagram depicting differentially expressed transcripts across cardiac chambers during development. Venn diagrams are color coded to represent a compartment of the heart: Yellow - Ventricle; Red - Atrium; Purple - Right Ventricle; Green - Left Ventricle; and Blue - Left Atrium. The diagram was created from E9.5 to P0, since P3, P7 and P21 only had left ventricle cells; thus, the compartment’s comparisons were not possible in these 3-time points. The number of transcripts is noted in the diagram through 4 different cell-types (Cardiomyocytes; Endothelial; Fibroblasts and Myofibroblasts; Macrophages and Monocytes) represented by each column. **A** This analysis shows the number of shared and unique coding transcripts of each compartment. **B** This analysis shows the number of shared and unique non-coding transcripts (lncRNA and pcRNA) of each compartment.

To understand the dynamics of transcript markers during myocardial development, we compared the expression patterns of cell-type specific markers across cardiac chambers. To achieve this, we evaluated the presence/absence of each marker of the four most abundant cell populations (cardiomyocytes, endothelial cells, fibroblasts, macrophages, and monocytes) per time during the course of myocardial development (Fig. 4). Our analysis unveiled distinct coding and non-coding RNA (lncRNAs, pcRNAs) signatures for the same cell-type in different chambers, suggesting the same cell-type adopts novel compartment-specific gene expression patterns per anatomical milieu. The proportions of shared and unique transcripts were similar between coding and non-coding profiles across anatomical areas and time-points, suggesting no bias towards one transcript type being more specific to a particular anatomical origin.

### Differential expression and functional enrichment analysis explored cardiomyocyte subpopulations

**Fig. 5:**
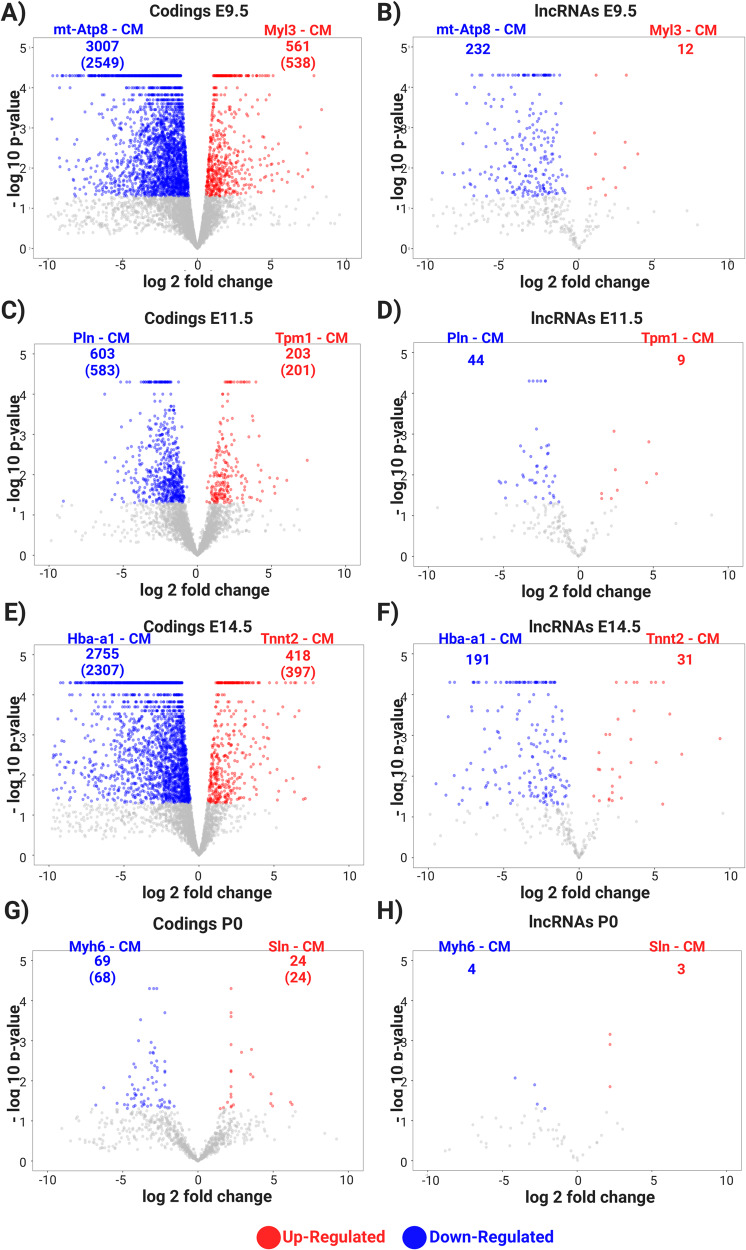
Volcano plots depicting cardiomyocyte subpopulations during the course of heart development. The left side (**A**, **C**, **E**, **G**) are related to coding transcripts in ventricle E9.5, left ventricle E11.5, right ventricle E14.5 and left atrium P0, respectively. The right side (**B**, **D**, **F**, **H**) are related to lncRNAs transcripts in ventricle E9.5, left ventricle E11.5, right ventricle E14.5 and left atrium P0, respectively. The blue and red colors are related to down- and up-regulated transcripts, respectively; the numbers are related to differentially expressed transcripts in each condition (down- and up-regulated); and the numbers in parenthesis are the number of differentially expressed genes.

To pursue differences within the identified cardiomyocyte subpopulations, we analyzed differential expression to characterize biological variation between the two subpopulations (Cardiomyocyte 1 and 2) identified in each chamber and developmental stage, using DESeq2 [29] and Volcano plot for data visualization. Different cardiomyocyte cell-types were identified in distinct chambers and time-points (Fig. 5), with the ventricle population of cells presenting greater number of differentially expressed (DE) genes across both cardiomyocyte types, with 3,568 coding and 244 lncRNAs detected as DE at embryonic stage E9.5 (ventricle); 806 coding and 53 lncRNAs in the embryonic stage E11.5 (left ventricle); and 3173 coding and 222 lncRNAs at E14.5 (right ventricle). We also identified distinct cardiomyocyte subpopulations in the left atrium at the post-natal time-point P0, with 84 coding and 7 lncRNAs identified as DE (Supplementary File 2). In summary, the ventricle population presents a greater number of DE genes and a set of DE genes were identified in each chamber and developmental stage.

To facilitate the identification of specific subpopulations, we named the cardiomyocytes by adding at the front of the cell type (e.g. cardiomyocyte (CM)) the name of the coding gene with the highest expression for each chamber and time-point. The ventricle E9.5 subpopulations were named “Mt-Atp8-CM” and “Myl3-CM”; left ventricle E11.5 were named “Pln-CM” and “Tpm1-CM”; right ventricle E14.5 as “Hba-a1-CM” and “Tnnt2-CM”; and the left atrium P0 were named “Mth6-CM” and “Sln-CM for P0”. All these genes have been described in the literature as involved in multiple mitochondrial and cardiovascular key processes and diseases. The blue and red colors are related to down- and up-regulated transcripts, respectively, relative to one another; the numbers are related to DE transcripts in each condition (down- and up-regulated); and the numbers in parenthesis are the number of DE lncRNAs, when presenting fold-change and *p* value cut-offs of 1.5 and 0.05, respectively. Notably, in all four time-points one cardiomyocyte subpopulation tended to dominate with a higher number of positively expressed gene transcripts compared to another for both the coding and lncRNA transcripts, except for lncRNAs P0 where the abundance was nearly equal. This suggests distinct gene expression profiles between the subpopulations.

**Fig. 6:**
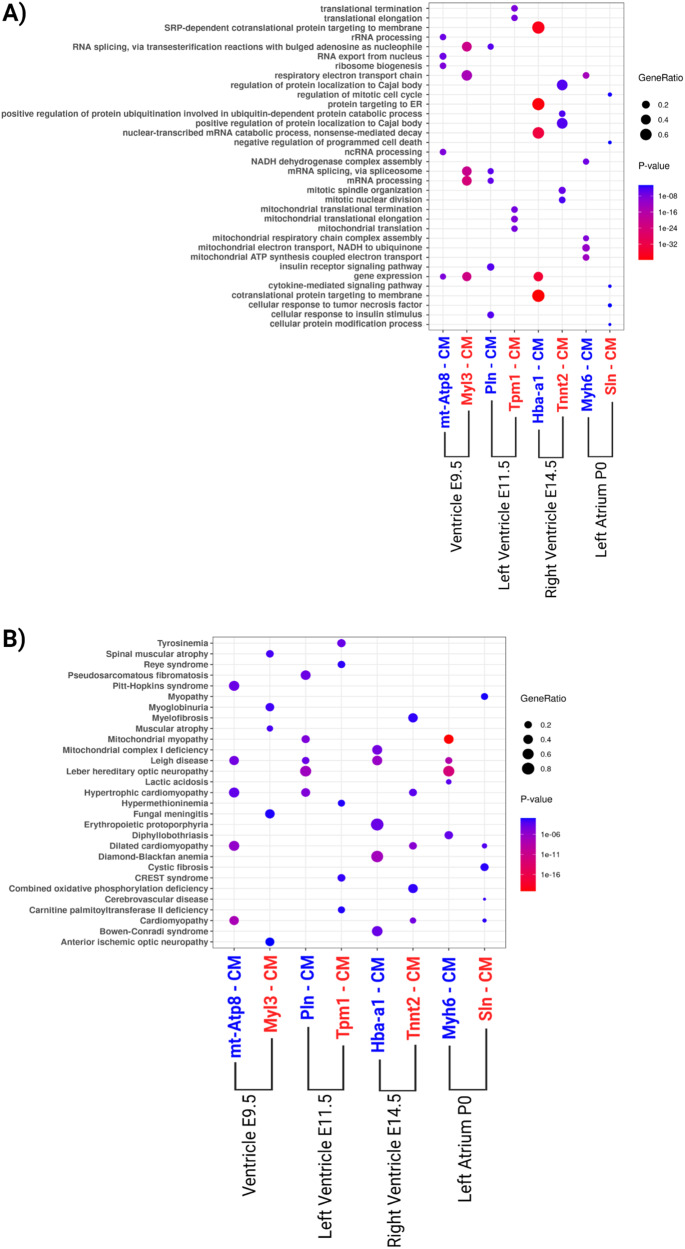
Enrichment of biological processes based on Gene Ontology (GO) pathway analysis and diseases in which the differential expressed genes are involved. The gene ratio size was computed through the number of overlap (observed/expected) genes. We named the cardiomyocyte’ populations according to the coding gene with higher expression in each chamber and time point. **A** Biological processes from GO analysis of each cardiomyocyte subpopulation. **B** Diseases enrichment analysis of each cardiomyocyte subpopulation according to Jansen Disease Database. The blue and red colors are related to down- and up-regulated transcripts, respectively.

To gain insights into the functional characteristics and potential disease associations of the DE transcripts, we performed enrichment analysis through EnrichR [30], using a corrected p-value cut-off of 0.05, and considering the databases Gene Ontology (GO) Biological Process [31], Jensen Diseases [32] and KEGG pathways [33] (Fig. 6 and Supplementary File 3). Figure 6 represents the GO Biological Processes and Jensen Diseases for each cardiomyocyte subpopulations top five terms. In the E9.5 ventricle, cell populations manifested associations with RNA processing and regulation (Fig. 6A). “Mt-Atp8-CM” cardiomyocytes manifested enrichment in transcripts related to cardiomyopathies and neurological disorders known to be associated with cardiomyopathies and heart defects. The “Myl3-CM” cardiomyocytes are enriched in transcripts associated with muscular diseases such as spinal muscular atrophy, myelofibrosis, and anterior ischemic optic neuropathy (Fig. 6B).

Regarding the left ventricle E11.5, the “Pln-CM” subpopulation was similarly associated with RNA processing, but also with GO terms linked to the insulin receptor signaling pathway (Fig. 6A). It was also enriched with genes related to hypertrophic and mitochondrial myopathies, pseudosarcomatous fibromatosis, Leigh disease, and Leber hereditary optic neuropathy (Fig. 6B). The “Tpm1-CM” subpopulation is enriched with transcripts associated with mitochondrial translational termination and elongation (Fig. 6A), and with different metabolic disorders such as tyrosinemia, Reye syndrome, hypermethioninemia, CREST syndrome, and carnitine palmitoyltransferase II deficiency (Fig. 6B).

In the right ventricle, the E14.5 “Hba-a1-CM” subpopulation was enriched with genes related to protein targeting the membrane and the endoplasmic reticulum (Fig. 6A). They were also associated with Leigh disease, as well as with blood-related diseases, mitochondrial complex I deficiency, and Bowen-Conradi syndrome. “Tnnt2-CM” cardiomyocytes manifested enrichment in genes associated with the regulation of protein localization to the Cajal body, regulation of protein ubiquitination, mitotic spindle organization, and nuclear division. They are associated with myelofibrosis, cardiomyopathies, and combined oxidative phosphorylation deficiency (Fig. 6B).

P0 left atrium cardiomyocytes “Myh6-CM” manifested enrichment in genes associated with mitochondrial function and respiratory electron transport chain (Fig. 6A), with a direct link with mitochondria-related diseases (Fig. 6B). The “Sln-CM” subpopulation was enriched with genes associated with the cell cycle, protein modification process, and cytokine-mediated signaling pathway (Fig. 6A). They were also linked with cardiomyopathies, besides cystic fibrosis, and cerebrovascular disease (Fig. 6B).

In summary, our differential expression and enrichment analyses uncovered distinct cardiomyocyte subpopulations with unique gene expression profiles and functional characteristics. These findings provide novel insights into the roles of different subpopulations in disease and contribute to a better understanding of cardiomyocyte heterogeneity in different chambers and developmental stages. Supplementary File 3 contains additional information on enriched GO terms, KEGG pathways and Jensen Diseases associated with each cardiomyocyte subpopulation.

### Modular expression analysis reveals cell-specific functional insights for lncRNAs during myocardial development

We decided to investigate whether transcriptional modules based on co-expression patterns can shed light on novel functional connections between lncRNAs and mRNAs [34] during heart development in a single-cell perspective. We employed CEMiTool to identify expression modules at each time-point, derive GO terms over-represented in each module, retrieve protein-protein and co-expression interactions within module transcripts and perform cell-specific gene set enrichment analysis (GSEA) [35] to identify the functional significance of the co-expressed gene modules in a subpopulation in heart development. Each module represents a group of transcripts that exhibit similar expression across cell-types, and the interactions enable us to explore for co-regulation among the genes. CEMiTool was able to generate modules for 13 out of 15-time points and heart compartments, without being able to retrieve modular patterns based on guilt-by-association for atrium E9.5 and left ventricle E14.5 (Supplementary File 3).

**Fig. 7:**
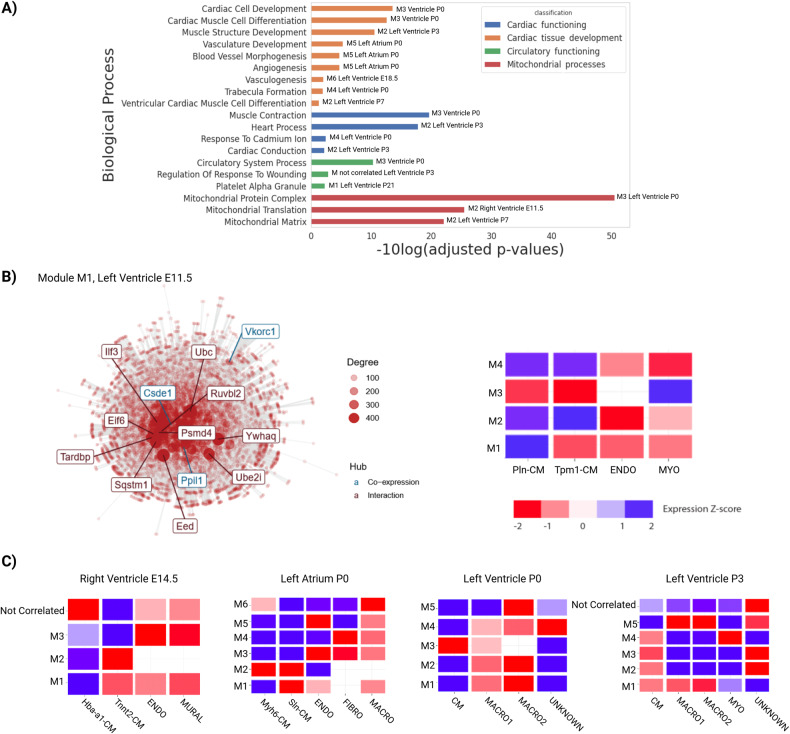
Modular expression analysis reveals cell-specific functional insights for lncRNAs along with heart development. **A** Biological processes from GO analysis of the modules that are related to the development of cardiac tissue, mitochondrial processes, cardiac functioning, and circulatory functioning. We observe the compartment and time point to which the adjusted p-value corresponds. **B** Module 1 (M1) from the Left ventricle E11.5 is enriched in mitochondrial processes and GSEA shows a difference between cardiomyocyte subpopulations 1 and 2. The co-expression network shows the gene´s interaction based on their expression patterns and protein-protein interaction, the gene’s names that appear are the hub genes that have more connectivity with others. **C** GSEAs were undertaken separately for each time point and heart compartment, revealing a cell-specific expression pattern for some of these modules. The blue and red colors are related to down- and up-regulated transcripts, respectively.

Once the modules were identified, we examined their enrichment in distinct cell-types and functional processes (Fig. 7A–C). Figure 7A illustrates the associations between the enriched modules and specific cell types or functional categories, highlighting modules related to cardiac tissue development, mitochondrial process, cardiac functioning, and circulatory function. Notably, Module 1 (M1) from the left ventricle E11.5 is enriched in mitochondrial processes, and the GSEA analysis shows a clear difference in the module expression between cardiomyocyte subpopulations Pln-CM and Tpm1-CM (Fig. 7B). GSEAs were undertaken separately for each time-point and heart compartment, revealing cell-specific expression patterns for some of these modules (Fig. 7C). This latter analysis revealed modules harboring different expression patterns in cardiomyocyte and macrophage subpopulations (Supplementary File 4). In summary, we identified specific modules that were differentially enriched in distinct functional processes, cell-types and subpopulations.

**Fig. 8:**
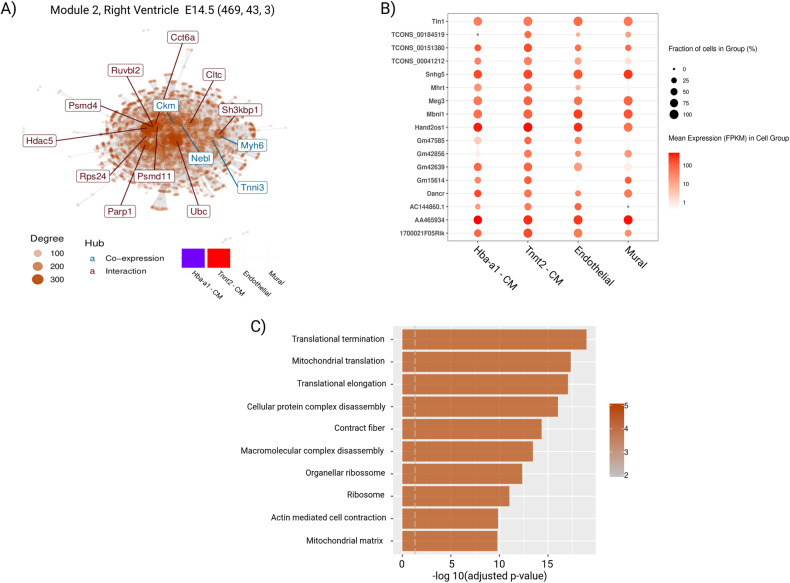
Expression of lncRNAs available in Module 2 from right ventricle E14.5. **A** Co-expression and protein-protein interaction network from Module 2, right ventricle samples from E14.5. The small heatmap shows the GSEA analysis. The numbers 469, 43 and 3 are related to the number of coding, lncRNAs and pcRNAs that compounds in this module. **B** The cell’s fraction and FPKM mean expression of 17 selected lncRNAs which compound the module. **C** Represents the top 10 GO terms based on the module’s enrichment analysis.

By the findings above, we selected enrichment analysis and modules directly related to the development of cardiac tissue. Figure 8A focuses on right ventricle samples from E14.5, and demonstrates a mitochondrial module with two cardiomyocyte subpopulations, with Hba-a1-CM being under-represented and Tnnt2-CM over-represented in the module. Figure 8B highlights some lncRNAs that are part of the module depicted in Fig. 8A. Some of these lncRNAs have been previously reported as directly involved in heart processes [2, 36–44]. Figure 8C presents the module’s enrichment analysis according to GO Biological Processes, providing valuable information about the functional categories associated with the genes within the specific module.

We observed *Tln1* FPKM expression means of 42.7 and 47.55 in Hba-a1-CM and Tnnt2-CM, respectively, while endothelial cells exhibited an expression at 70.72 (Fig. 8). *Tln1* showed low expression levels in normal CMs compared to other cell-types. Additionally, we found a *Snhg5* FPKM expression mean of 265.8 in mural cells, greater than the other cell-types. This pcRNA contributes to angiogenesis in acute myelogenous leukemia (Supplementary File 4).

Notably, cardiomyocyte subpopulations were observed in specific stages and compartments of myocardial development, and these subpopulations manifested distinct co-expression patterns, as revealed by t-SNE plots and heatmaps (e.g. Fig. 2, Supplementary Fig. 1). Clusters classified as “unknown” (left ventricle P0 and left ventricle P3) manifested unique co-expression behaviors compared to other types in the same condition. These cells likely represent another cell type for which specific markers have not been identified. Based on the markers defined by the M3Drop, only one was determined for these unknown cells (Qk—left ventricle P0; and ENSMUST00000118613.6—left ventricle P3).

Previous studies have described cardiomyocyte subpopulations [45] and lncRNAs associated with these subpopulations [45, 46]. Notably, *Gas5* and *Sghrt* (previously annotated as *1810058i24Rik*) have been implicated in cardiac hypertrophy in a rat model [46], and certain regulatory lncRNAs appear to influence cell cycle arrest [45]. We also detected that these two lncRNAs are DE throughout heart development and cardiomyocyte subpopulations (Supplementary File 2).

## Discussion

Gene expression is a fundamental mechanism for transmitting genetic information [1]. Gene expression encyclopedias catalog genes that are co-expressed in different cell-types, facilitating the understanding of their functions and regulatory processes [6]. LncRNAs comprise the most representative transcriptional units in mammalians and are associated with different organs development like corpus luteum formation, heart, skeletal muscle, brain and certain diseases [11, 47–49]. The study of heart development is of significance, given the toll taken by cardiovascular diseases.

We identified eight different cell types from different regions and stages of heart development, more than that described in the original scRNA-seq study, but the authors discuss that it would be possible to obtain additional cell lineages by improving the analysis of the dataset they obtain [20]. Interestingly, the fibroblasts and myofibroblasts transcriptional signatures were detected at E14.5, the stage at which fibroblasts from the epicardium are known to undergo EMT into the myocardium [26]. Cell populations with monocytes and macrophages profiles appeared only at late embryonic and postnatal stages, consistent with evidence in the literature of monocyte-derived macrophages increasing in the myocardium post-birth [27].

New marker transcripts distributed in coding genes and lncRNAs were identified, in addition, some found specifically in certain compartments, such as *Myl3* that is known as a ventricular light chain myosin with increased expression in slow-twitch skeletal muscle fibers; and *Sln*, which is restricted to the atrial lineage during mouse heart development. Other transcripts are shared between compartments such as *Tpm1* with established roles in cardiac looping, atrial septation, and ventricular trabeculae formation.

Different cardiomyocyte subpopulations were identified in distinct chambers and time-points (E9.5, E11.5, E14.5 and P0), with the ventricle and left atrium presenting greater differences within both cardiomyocyte types. We named the cardiomyocyte populations according to the coding gene with higher expression in each chamber and time-point. Of note, some of these genes are associated with heart diseases, *Mt-Atp8*, for example, is enriched in cardiomyocytes and is involved in many diseases in the ventricle and neuropathies [47]. *Myl3* was classified as a cardiac protein that was abundantly expressed in the heart (>260 TPM) with mosaic patterns of expression in skeletal muscle [48]. Additionally, *Myl3*, known as a ventricular light chain myosin with increased expression in slow-twitch skeletal muscle fibers, is robustly and homogeneously present in ventricular cardiomyocytes [48]. *Myl3* was described as DE in the ventricle and is associated with muscle atrophy and neuromuscular diseases [49]. A study in a mouse model of Huntington’s disease also reported elevated serum levels of *Myl3* concomitant with skeletal muscle atrophy [50].

*Pln*, overexpressed in the left ventricle and called cardiac phospholamban, modulates calcium re-uptake during muscle relaxation and plays an essential role in calcium homeostasis in the heart [51]. Moreover, mutations in *Pln* have been associated with dilated cardiomyopathy (DCM), hypertrophic cardiomyopathy (HCM) [52], and arrhythmogenic right ventricular cardiomyopathy (ARVC) [52]. *Tpm1* plays multiple roles in heart development and the formation of congenital heart defects [53]. Mutations in *Tpm1* have been associated with both HCM, DCM [53, 54], and with peripartum cardiomyopathy (PPCM) [54].

*Hba* − *a1* is up-regulated in response to chronic stress along with genes associated with the vascular system. A reduced expression of *Hba* − *a1* is related to a neuroprotective role [55]. *Tnnt2* is a cardiac muscle troponin T that is expressed throughout heart development and in the post-natal heart, and in the left and right ventricle [56]. *Tnnt2* is also associated with hypertrophic cardiomyopathy, dilated cardiomyopathy, and restrictive cardiomyopathies [56]. *Myh6* is a cardiac muscle myosin that is preferentially expressed in the atrial chambers and its mutation causes atrial septal defect [57]. *Myh6* was described in differentiation in adult cardiac precursor cells (CPCs), which were characterized by up-regulation of *Myh6* and, therefore, massively differentiated into cardiomyocytes [58]. Studies have also reported additional mutations in *Myh6* linked to both hypertrophic cardiomyopathy and dilated cardiomyopathy [59].

The expression of *Sln* is restricted to the atrial lineage during mouse heart development, and this pattern is conserved in other mammals, including humans [60]. *Sln* is involved with Ca^2+^ handling that plays a crucial role in the contraction and relaxation of cardiomyocytes [60]. Moreover, *Sln* is involved with dilated cardiomyopathy, cardiomyopathy [61], and heart failure [60].

Our examination revealed transcriptional gene modules related to the development of cardiac tissue, mitochondrial processes, cardiac functioning, and circulatory functioning. Our cardiomyocyte populations were enriched in mitochondrial functions, but we also identified differences between cardiomyocyte subpopulations according to GSEA. We identified some lncRNAs that are already described in the literature as directly related to heart processes: *Mhrt* is involved with cardiac hypertrophy and heart failure [36, 37], and also protects cardiomyocytes against H_2_O_2_-induced apoptosis [38]; *Tln1* is expressed at low levels in normal CMs compared with other cell-types, and combined deletion of CMs *Tln1* and *Tln2* destabilized the myocardium, leading to heart failure [39]. Figure 8 demonstrates a *Tln1* FPKM expression mean of 42.7 and 47.55 in “Hba-a1-CM” and “Tnnt2-CM”, respectively, while in endothelial cells, we observed expression at 70.72, evidencing a cell-specific higher expression pattern. *Hand2os1* orchestrates heart development; its locus dampens *Hand2* expression to restrain cardiomyocyte proliferation, thereby orchestrating balanced development of cardiac cell lineages [(40)]; *Dancr* is a lncRNA that has coding potential in the human heart, encoding putative micropeptides and is associated with cardiovascular diseases [41]; *Mbnl1* regulates isoproterenol‐induced myocardial remodeling in vitro and in vivo [62]; *Meg3* prevents cardiac fibrosis and diastolic dysfunction [2, 62]; *AA465934* is associated with early diabetic cardiomyopathy [42]. Finally, *Snhg5* upregulation induced by YY1 contributes to angiogenesis in acute myelogenous leukemia [63]. We observed a *Snhg5* FPKM expression mean of 265.8 in mural cells, greater than the other cell-types (Fig. 8). Mural cells are integral components of brain blood vessels that play important roles in vascular formation, regulation of regional cerebral blood flow, regulation of vascular stability, and homeostasis [64]. *Snhg5* has also been reported to participate in the occurrence and development of glioma [44].

However, even though we can describe different subpopulations of cardiomyocytes, there are some limitations. These subpopulations could only be identified in certain chambers of the heart and in some stages of development. Experimental techniques such as Fluorescence-activated Cell Sorting (FACS) of cell surface markers or in situ hybridization with specific transcripts will need to be used to individually isolate these two potential cardiomyocyte subpopulations. However, using single cell and hierarchical clustering strategies allows the identification of possible cell-types in other tissues during development.

Our results evidence the role of lncRNAs in heart development and highlights the usage of co-expression modular approaches in the cell-type functional definition. Perspectives of the present work are: knowledge generated here unveils potential processes guided by these lncRNAs during cardiac differentiation or development, which can lead to the development of improved therapeutics for cardiovascular diseases targeting genes potentially involved with the re-expression of the fetal gene expression program. In our analysis, several coding and non-coding transcripts were identified, and they can be used as early biomarkers for the prognosis for several cardiomyopathies, for example, *Mhrt* in cardiac hypertrophy and heart failure, *Meg3* in cardiac fibrosis, *Snhg5* in vascular cerebral homeostasis, among others. Then, future studies should investigate how these lncRNAs regulate other RNAs, like miRNAs, in cardiac development and diseases.

## Methods

The online version contains supplementary detailed methods available at X.

### Supplementary information

Supplementary Methods

Supplementary Figure Legends

Suppl Figure 1

Suppl Figure 2

Suppl File 1

Suppl File 2

Suppl File 3

Suppl File 4

Author Contributions

Checklist

## Data Availability

The authors declare that all single-cell sequencing data reported in this article have been deposited are available within the paper, Supplementary Information files and deposited in the GNomEx database (https://b2b.hci.utah.edu/gnomex/) under accession numbers 272 R, 274 R, 275–292 R, 439 R, and 440 R.
