# Supplementary material for: Single-cell transcriptional landscape of long non-coding RNAs orchestrating mouse heart development
Source: Cell Death Dis. 2023 Dec 18;14(12):841. doi: 10.1038/s41419-023-06296-9 (PMC10728149; doi:10.1038/s41419-023-06296-9)
Supplement: Supplementary file 1 — Supplementary Methods [file 41419_2023_6296_MOESM1_ESM.docx]

***Supplementary Information: Detailed Methods***

**Single-cell transcriptional landscape of long non-coding RNAs orchestrating mouse heart development**

**Thaís A. R. Ramos^1,2,3^, Sebastián Urquiza-Zurich^1^, Soo Young Kim^4^,**

**Thomas G. Gillette^4^, Joseph A. Hill^4,5^, Sergio Lavandero^1,6,*^,**

**Thaís G. do Rêgo^2,3,*^ and Vinicius Maracaja-Coutinho^1,2,*^**

*^1^ Advanced Center for Chronic Diseases (ACCDiS), Faculty of Chemical & Pharmaceutical Sciences & Faculty of Medicine, Universidad de Chile, Santiago, Chile.*

*^2^ Programa de Pós-Graduação em Bioinformática, Bioinformatics Multidisciplinary Environment (BioME), Instituto Metrópole Digital, Universidade Federal do Rio Grande do Norte, Brazil.*

*^3^ Departamento de Informática, Centro de Informática, Universidade Federal da Paraíba, João Pessoa, Brazil.*

*^4^ Division of Cardiology, Department of Internal Medicine, University of Texas Southwestern Medical Center Dallas, Texas, USA.*

*^5^ Department of Molecular Biology, University of Texas Southwestern Medical Center, Dallas, Texas, USA.*

*^6^ Corporación Centro de Estudios Científicos de las Enfermedades Crónicas (CECEC), Santiago, Chile.*

**METHODS**

**Datasets selection and filtering criteria**

We used a cardiac single-cell RNA-seq dataset from *Mus musculus* model organism provided by DeLaughter *et al.* [[1](https://paperpile.com/c/wGBUhw/qQDwd)]. The dataset was generated from 1,695 single-cells from 4 embryonic (E9.5, E11.5, E14.5, E18.5) and 4 post-natal (P0, P3, P7, P21) stages, according to 5 different regions of the heart (ventricle, atrium, left ventricle, right ventricle, and left atrium), captured using the Fluidigm integrated fluidic circuits (IFC). For the lncRNA reference database, we combined lncRNAs from Gencode (M20), Ensembl (GRCm38.95) and Amaral *et al.* (2018) [[2](https://paperpile.com/c/wGBUhw/oXDnf)], which contained 18,339, 9,074 and 15,757 lncRNAs, respectively. For coding transcripts, we selected the Gencode (M20) dataset. Since the lncRNAs reference dataset was built from 3 different databases, preprocessing was performed to remove the redundant sequences and generate the final dataset. In this process, we eliminated the lncRNAs with more than 50% of overlap [[2](https://paperpile.com/c/wGBUhw/oXDnf)] from one database to another, resulting in the final dataset of 21,044 lncRNAs. To obtain expression levels of lncRNAs and coding transcripts we performed the following pipeline: Fastqc version 0.11.9, to execute the quality control [[3,4](https://paperpile.com/c/wGBUhw/u5rgI+zbZc)]; Trimmomatic version 0.39, to trim and crop the bases with low quality as well as to remove the adapters [[5](https://paperpile.com/c/wGBUhw/o8rVQ)] (parameters: 2:30:10 LEADING:5 TRAILING:5 SLIDINGWINDOW:4:28 MINLEN:32); Hisat2 version 2.1.0, to mapping sequencing reads [[6](https://paperpile.com/c/wGBUhw/w5mUQ)], using --dta-cufflinks parameter to makes Hisat2 alignment tailored specifically for Cufflinks; Samtools version 1.3.1, to convert SAM to BAM and sort the BAM's files [[7](https://paperpile.com/c/wGBUhw/93cF5)]; Cuffnorm, part of Cufflinks package version 2.2.1, to report expression levels normalized by FPKM [[8](https://paperpile.com/c/wGBUhw/qJTo)]. To determine expressed transcripts and its filtering to avoid sparsity issues, we used a filter of an expression value (FPKM > 0.001) [[2](https://paperpile.com/c/wGBUhw/oXDnf)] and transcript expression percentage in cells: transcript had to be expressed in at least 5% of the cells [[9,10](https://paperpile.com/c/wGBUhw/OZQbM+dwC8E)]. Then, we performed transcriptome saturation at a random stage of the heart development (left ventricle at E18.5) to show that lncRNAs are identified with this pipeline (Supplementary Fig. 2).

##

## **Cell clustering**

## We utilized the M3Drop R package [[11](https://paperpile.com/c/wGBUhw/JqZTq)], which fits a Michaelis-Menten model to the pattern of dropouts in single-cell RNA-seq data. This model was used to identify significantly variable (*i.e*. differentially expressed) genes to clusterize cells. Next, we utilized the Silhouette method [[12](https://paperpile.com/c/wGBUhw/lTIQ7)] to estimate the best clusters and performed unsupervised hierarchical clustering with Ward linkage from scikit-learn Python library to group the cells. Then, we performed Principal Component Analysis (PCA) to reduce the dimensionality and t-distributed Stochastic Neighbor Embedding (t-SNE) for better visualization of the clusters with high dimensionality.

## **Gene markers, cell types identification, and clustering assignment**

## We generated a cell marker database using gene markers from DeLaughter *et al.* [[1](https://paperpile.com/c/wGBUhw/qQDwd)], Gladka *et al.* [[13](https://paperpile.com/c/wGBUhw/KHKqR)], Farbehi *et al.* [[14](https://paperpile.com/c/wGBUhw/hIma5)], and Franzén *et al.* [[15](https://paperpile.com/c/wGBUhw/rwpmr)]. Markers that were classified for more than one cell type were eliminated. The number of final gene markers for each cell type was: cardiomyocytes (70); endothelial cells (100); fibroblasts and myofibroblasts (140); macrophages and monocytes (128); B-cells (76); dendritic cells (42); glial cells (2); natural killer cells (35); T-cells (59); mural cells, vascular smooth muscle cells and pericytes (42) and myoblasts (16). Using this cell marker unified database, we checked the frequency of appearance of these genes in cardiac single cell samples from DeLaughter *et al*. [[1](https://paperpile.com/c/wGBUhw/qQDwd)] and saw that there was no significant appearance of markers from glial cells and natural killer cells and eliminated these 2 cell types. Our final dataset was composed of markers for 9 cell types, which were used to assign cell types to the clusters: cardiomyocytes; endothelial; fibroblasts and myofibroblasts; macrophages and monocytes; B-cells; dendritic; T-cells; mural cells; vascular smooth muscle cells and pericytes; and myoblasts. M3Drop [[11](https://paperpile.com/c/wGBUhw/JqZTq)] was used to find the marker transcripts of each cluster and chi-squared and adherence tests were used to determine the cluster's assignment signification (*i.e*. p-value < 0.05).

##

## **Co-expression modular analysis**

## To get the knowledge of genes and lncRNAs that are co-expressed and to understand the biological processes linked we used CEMiTool [[16](https://paperpile.com/c/wGBUhw/Ic8Ml)], a systems biology method that identifies co-expression gene modules. We also used CEMiTool to perform comprehensive modular analyses, including: gene set enrichment analysis (GSEA) within samples, modules and cell types; functional enrichment analysis of modules (using Supplementary Dataset 1); and integrate co-expression results with protein-protein interaction data (using Supplementary Dataset 2). It allowed us to identify functional categories of protein-coding genes co-expressed with lncRNAs and identify those lncRNAs which were associated with the modules that were enriched in heart development processes.

## **Cardiomyocyte sub-populations differential expression and functional enrichment analysis**

## To identify the biological variation between cardiomyocyte sub-populations, we performed differential expression (DE) analysis among sub-populations in each time point and chamber in which two types of cardiomyocytes were identified: ventricle E9.5; left ventricle E11.5; right ventricle E14.5; and left atrium P0. DE transcripts were determined using Cuffdiff, from Cufflinks [[17](https://paperpile.com/c/wGBUhw/dqWA1)]. Transcripts were considered as statistically DE when presenting fold-change and p-value cut-offs of 1.5 and 0.05, respectively. Functional overrepresentation analyses of DE transcripts were performed using different databases available in the EnrichR web tool [[18](https://paperpile.com/c/wGBUhw/PIeMp)], using a corrected p-value cut-off of 0.05, and considering the databases Gene Ontology Biological Process [[19](https://paperpile.com/c/wGBUhw/uBd5S)], KEGG pathways [[20](https://paperpile.com/c/wGBUhw/ZUDSy)] and Jensen Diseases [[21](https://paperpile.com/c/wGBUhw/C8Fy)].

**REFERENCES**

1. [DeLaughter DM, Bick AG, Wakimoto H, McKean D, Gorham JM, Kathiriya IS, et al. Single-Cell Resolution of Temporal Gene Expression during Heart Development. Dev Cell. 2016 Nov 21;39(4):480–90.](http://paperpile.com/b/wGBUhw/qQDwd)

2. [Amaral PP, Leonardi T, Han N, Viré E, Gascoigne DK, Arias-Carrasco R, et al. Genomic positional conservation identifies topological anchor point RNAs linked to developmental loci. Genome Biol. 2018 Mar 15;19(1):32.](http://paperpile.com/b/wGBUhw/oXDnf)

3. [Babraham Bioinformatics - FastQC A Quality Control tool for High Throughput Sequence Data [Internet]. [cited 2019 Oct 29]. Available from:](http://paperpile.com/b/wGBUhw/u5rgI) <http://www.bioinformatics.babraham.ac.uk/projects/fastqc/>

4. [Andrews S. FastQC: a quality control tool for high throughput sequence data http://www. bioinformatics. babraham. ac. uk/projects/fastqc. J Exp Med.](http://paperpile.com/b/wGBUhw/zbZc)

5. [Bolger AM, Lohse M, Usadel B. Trimmomatic: a flexible trimmer for Illumina sequence data. Bioinformatics. 2014 Aug 1;30(15):2114–20.](http://paperpile.com/b/wGBUhw/o8rVQ)

6. [Kim D, Paggi JM, Park C, Bennett C, Salzberg SL. Graph-based genome alignment and genotyping with HISAT2 and HISAT-genotype. Nat Biotechnol. 2019 Aug 2;37(8):907–15.](http://paperpile.com/b/wGBUhw/w5mUQ)

7. [Li H, Handsaker B, Wysoker A, Fennell T, Ruan J, Homer N, et al. The Sequence Alignment/Map format and SAMtools. Bioinformatics. 2009;25(16):2078–9.](http://paperpile.com/b/wGBUhw/93cF5)

8. [Trapnell C, Williams BA, Pertea G, Mortazavi A, Kwan G, van Baren MJ, et al. Transcript assembly and quantification by RNA-Seq reveals unannotated transcripts and isoform switching during cell differentiation. Nat Biotechnol. 2010;28(5):511–5.](http://paperpile.com/b/wGBUhw/qJTo)

9. [Saelens W, Cannoodt R, Todorov H, Saeys Y. A comparison of single-cell trajectory inference methods. Nat Biotechnol. 2019;37(5):547–54.](http://paperpile.com/b/wGBUhw/OZQbM)

10. [Carter RA, Bihannic L, Rosencrance C, Hadley JL, Tong Y, Phoenix TN, et al. A Single-Cell Transcriptional Atlas of the Developing Murine Cerebellum. Curr Biol. 2018;28(18):2910–20.e2.](http://paperpile.com/b/wGBUhw/dwC8E)

11. [Andrews TS, Hemberg M. M3Drop: dropout-based feature selection for scRNASeq. Bioinformatics. 2019 Aug 15;35(16):2865–7.](http://paperpile.com/b/wGBUhw/JqZTq)

12. [Rousseeuw PJ. Silhouettes: A graphical aid to the interpretation and validation of cluster analysis. J Comput Appl Math. 1987;20:53–65.](http://paperpile.com/b/wGBUhw/lTIQ7)

13. [Gladka MM, Molenaar B, de Ruiter H, van der Elst S, Tsui H, Versteeg D, et al. Single-Cell Sequencing of the Healthy and Diseased Heart Reveals Cytoskeleton-Associated Protein 4 as a New Modulator of Fibroblasts Activation. Circulation. 2018;138(2):166–80.](http://paperpile.com/b/wGBUhw/KHKqR)

14. [Farbehi N, Patrick R, Dorison A, Xaymardan M, Janbandhu V, Wystub-Lis K, et al. Single-cell expression profiling reveals dynamic flux of cardiac stromal, vascular and immune cells in health and injury. 2019](http://paperpile.com/b/wGBUhw/hIma5)

15. [Franzén O, Gan LM, Björkegren JLM. PanglaoDB: a web server for exploration of mouse and human single-cell RNA sequencing data. Database. 2019;2019](http://paperpile.com/b/wGBUhw/rwpmr).

16. [Russo PST, Ferreira GR, Cardozo LE, Bürger MC, Arias-Carrasco R, Maruyama SR, et al. CEMiTool: a Bioconductor package for performing comprehensive modular co-expression analyses. BMC Bioinformatics. 2018;19(1):56.](http://paperpile.com/b/wGBUhw/Ic8Ml)

17. [Trapnell C, Hendrickson DG, Sauvageau M, Goff L, Rinn JL, Pachter L. Differential analysis of gene regulation at transcript resolution with RNA-seq. Nat Biotechnol. 2012;31(1):46–53.](http://paperpile.com/b/wGBUhw/dqWA1)

18. [Kuleshov MV, Jones MR, Rouillard AD, Fernandez NF, Duan Q, Wang Z, et al. Enrichr: a comprehensive gene set enrichment analysis web server 2016 update. Nucleic Acids Res. 2016;44(W1).](http://paperpile.com/b/wGBUhw/PIeMp)

19. [Mi H, Muruganujan A, Ebert D, Huang X, Thomas PD. PANTHER version 14: more genomes, a new PANTHER GO-slim and improvements in enrichment analysis tools [Internet]. Vol. 47, Nucleic Acids Research. 2019. p. D419–26.](http://paperpile.com/b/wGBUhw/uBd5S)

20. [Kanehisa M, Goto S. KEGG: Kyoto Encyclopedia of Genes and Genomes. Nucleic Acids Res. 2000;28(1):27–30.](http://paperpile.com/b/wGBUhw/ZUDSy)

21. [Grissa D, Junge A, Oprea TI, Jensen LJ. Diseases 2.0: a weekly updated database of disease-gene associations from text mining and data integration. Database. 2022;2022.](http://paperpile.com/b/wGBUhw/C8Fy)
