# Supplementary material for: Single-cell transcriptional landscape of long non-coding RNAs orchestrating mouse heart development
Source: Cell Death Dis. 2023 Dec 18;14(12):841. doi: 10.1038/s41419-023-06296-9 (PMC10728149; doi:10.1038/s41419-023-06296-9)
Supplement: Supplementary file 2 — Supplementary Figure Legends [file 41419_2023_6296_MOESM2_ESM.docx]

***Supplementary Information: Supplementary Figures and Files Legends***

**Single-cell transcriptional landscape of long non-coding RNAs orchestrating mouse heart development**

**Thaís A. R. Ramos^1,2,3^, Sebastián Urquiza-Zurich^1^, Soo Young Kim^4^,**

**Thomas G. Gillette^4^, Joseph A. Hill^4,5^, Sergio Lavandero^1,6,*^,**

**Thaís G. do Rêgo^2,3,*^ and Vinicius Maracaja-Coutinho^1,2,*^**

*^1^ Advanced Center for Chronic Diseases (ACCDiS), Faculty of Chemical & Pharmaceutical Sciences & Faculty of Medicine, Universidad de Chile, Santiago, Chile.*

*^2^ Programa de Pós-Graduação em Bioinformática, Bioinformatics Multidisciplinary Environment (BioME), Instituto Metrópole Digital, Universidade Federal do Rio Grande do Norte, Brazil.*

*^3^ Departamento de Informática, Centro de Informática, Universidade Federal da Paraíba, João Pessoa, Brazil.*

*^4^ Division of Cardiology, Department of Internal Medicine, University of Texas Southwestern Medical Center Dallas, Texas, USA.*

*^5^ Department of Molecular Biology, University of Texas Southwestern Medical Center, Dallas, Texas, USA.*

*^6^ Corporación Centro de Estudios Científicos de las Enfermedades Crónicas (CECEC), Santiago, Chile.*

**Supplementary Figure 1.** Heatmaps and hierarchical clusters of the expression patterns for each cell type in each heart chamber and time-point across heart development.

**Supplementary Figure 2.** Number and fraction reads of coding and long non-coding RNAs in the left ventricle at E18.5 by transcriptome saturation in our pipeline.

**Supplementary File 1.** List of mRNAs, lncRNAs, and pcRNAs determined as transcript cell markers for each cell type.

**Supplementary File 2.** Differentially expressed coding genes and lncRNAs in the different cardiomyocyte subpopulations.

**Supplementary File 3.** Contains additional information about the EnrichR enrichment analysis considering the databases Gene Ontology (GO), Biological Process, Jensen Diseases and KEGG pathways associated with each cardiomyocyte subpopulation. All terms are named if they were upregulated (up) or downregulated (down) and followed by the time-point across heart development.

**Supplementary File 4.** Co-expressed modules identified for each developmental stage. Gene Set Enrichment Analysis, functional overrepresentation and interaction network (co-expression and protein-protein) are highlighted for each time point.
