# Supplementary figures and images for: Single-cell transcriptional landscape of long non-coding RNAs orchestrating mouse heart development
Source: Cell Death Dis. 2023 Dec 18;14(12):841. doi: 10.1038/s41419-023-06296-9 (PMC10728149; doi:10.1038/s41419-023-06296-9)

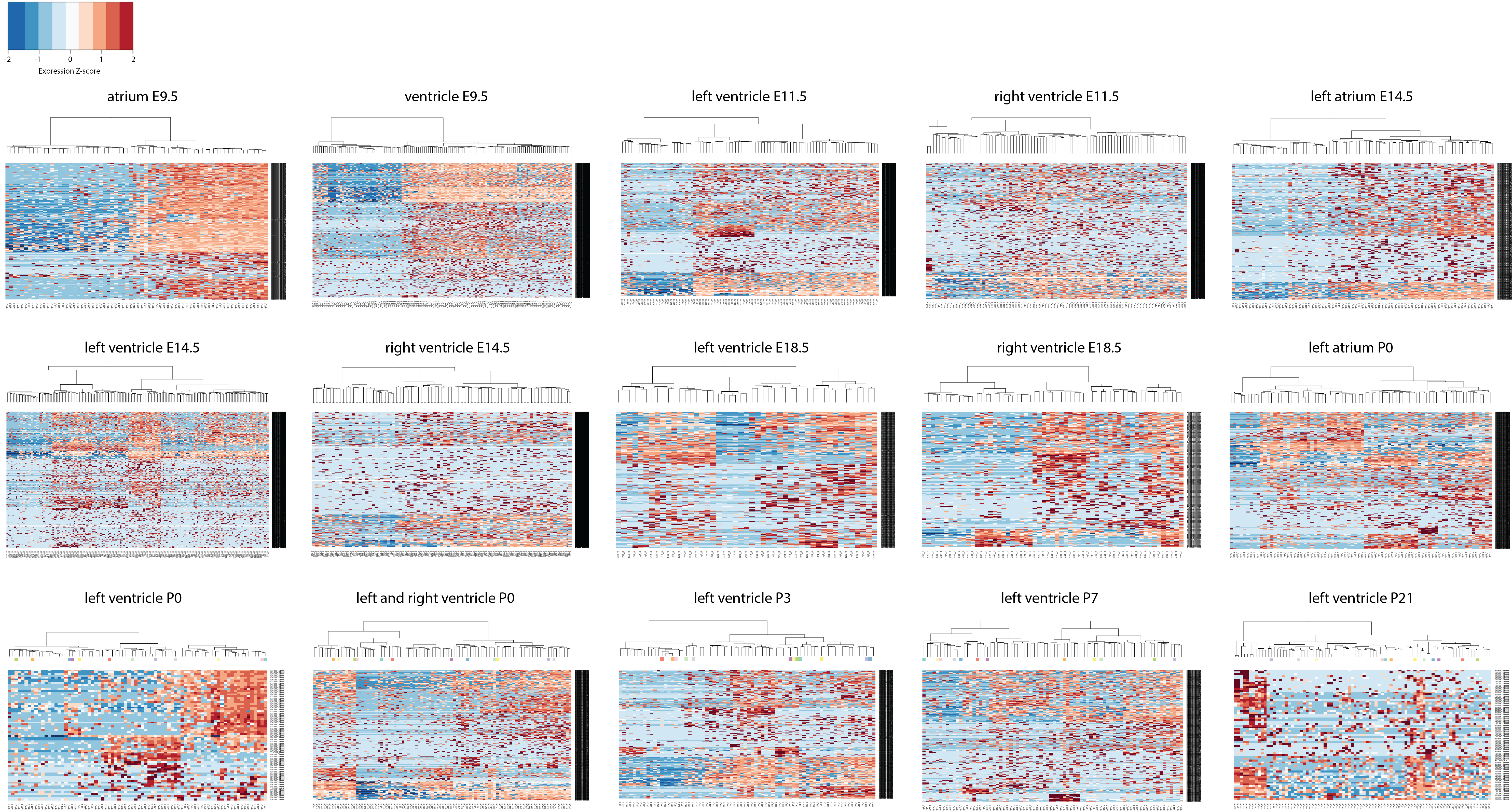

Supplement: Supplementary file 3 — Suppl Figure 1 [file 41419_2023_6296_MOESM3_ESM.png]

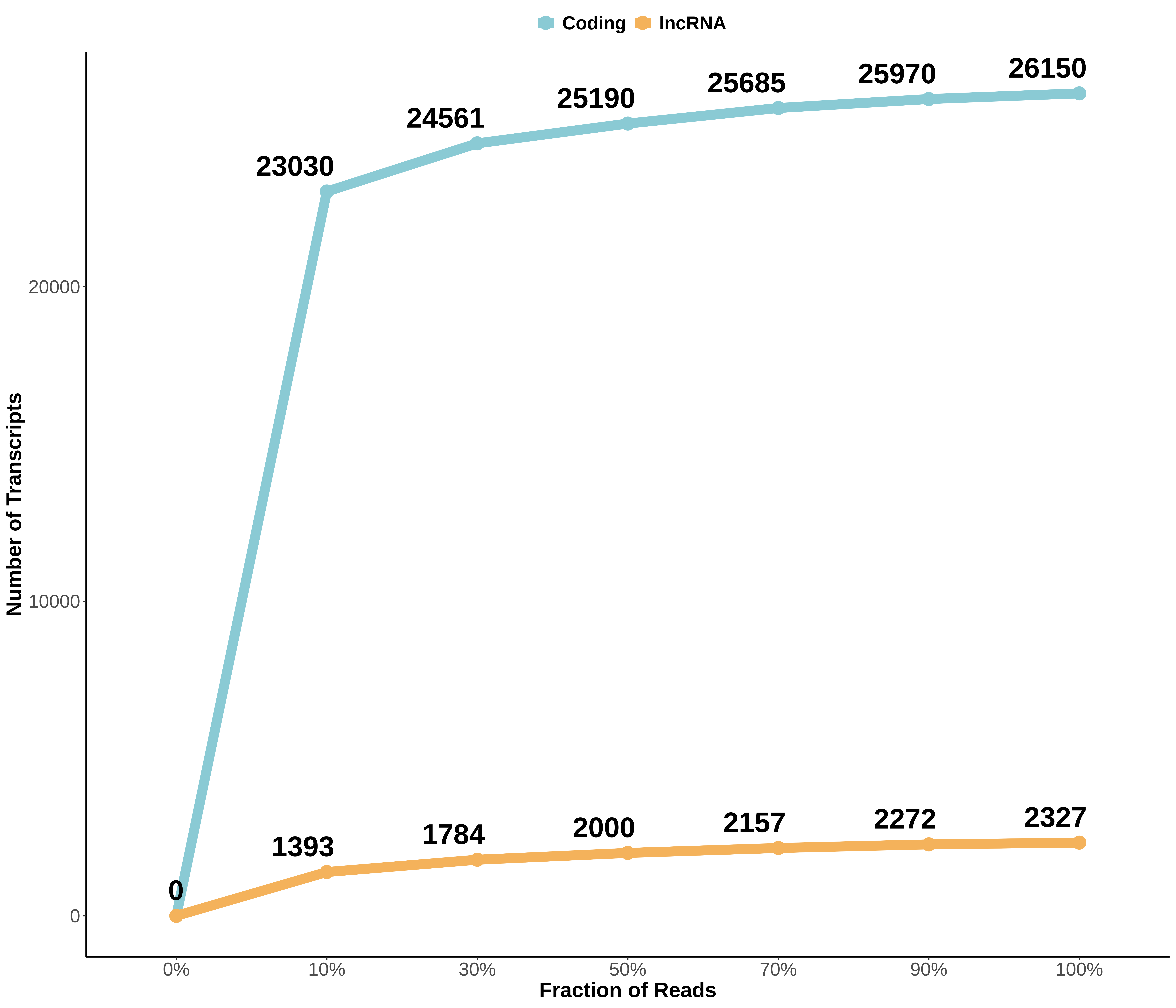

Supplement: Supplementary file 4 — Suppl Figure 2 [file 41419_2023_6296_MOESM4_ESM.png]

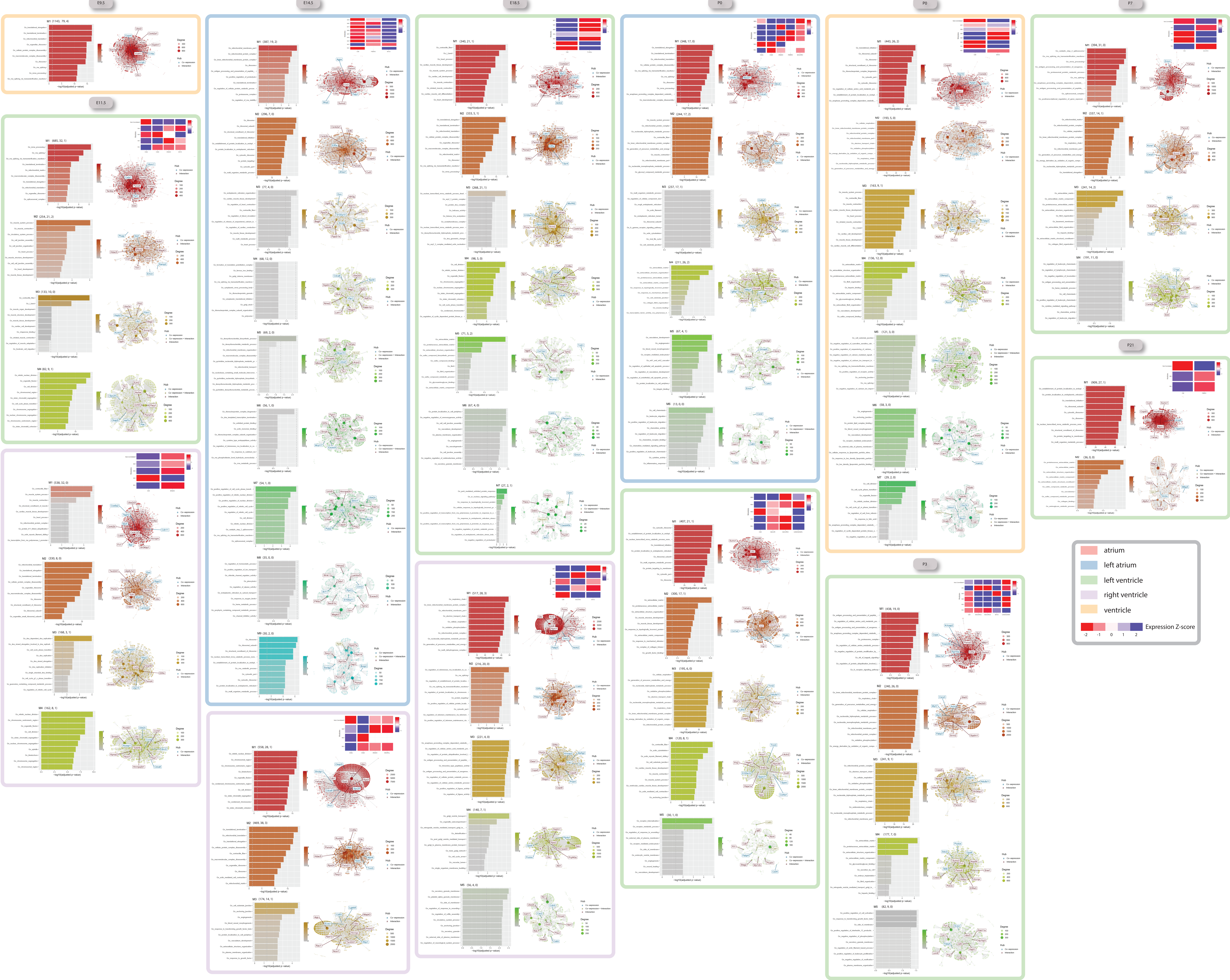

Supplement: Supplementary file 8 — Suppl File 4 [file 41419_2023_6296_MOESM8_ESM.pdf]
